# Methodological issues and controversies in research on cognitive disorders

**DOI:** 10.1590/S1980-57642010DN40400004

**Published:** 2010

**Authors:** Benito Pereira Damasceno

**Affiliations:** MD, PhD, Professor, Department of Neurology, Medical School, State University of Campinas (UNICAMP), Campinas SP, Brazil.

**Keywords:** cognition, neuropsychological tests, double dissociation, lesion localization, cognitive disorder research, epilepsy

## Abstract

Research on cognitive disorders is challenging due to the complexity of functions studied and to the numerous variables involved. First, the concept of cognition as a mediated (semiotic) and systemic activity is reviewed. According to this concept, the result of a local lesion is not an isolated symptom but a syndrome, and the best neuropsychological approach is an analysis based on appropriate tests to disclose double dissociations and thereby provide clues to brain-behavior relationships. This approach takes into account the influence of task-relevant variables (confounders) related to the patient (e.g., age, education), to the lesion (size, etiology), and to the tests and testing conditions (ecological validity, examiner’s experience), which need to be controlled and analyzed in multivariate statistical analyses, as illustrated in research on medial temporal lobe epilepsy. Other controversial issues such as single and double dissociations, single-case versus group studies, and the lesion method are also examined.

## Abstract (pt)

O estudo das desordens cognitivas é tarefa dificil devido à complexidade das funções estudadas e às numerosas variáveis implicadas. Neste artigo, nós primeiro revisitamos o conceito de cognição como atividade sistêmica e mediada (semiótica), de acordo com o qual a consequência de uma lesão focal não é um sintoma isolado mas uma syndrome, e a melhor abordagem neuropsicológica é uma análise baseada em testes apropriados para detectar dissociações duplas e, assim, fornecer pistas para relações cerebro-comportamentais. Esta abordagem leva em conta a influência de variáveis que podem influenciar o desempenho nos testes, as quais podem estar relacionadas ao paciente (idade, educação, etc.), à lesão (tamanho, etiologia), aos testes ou condições da testagem (validação ecológica, experiência do examinador), as quais precisam ser controladas e incluídas em análises estatísticas multivariadas, tal como fazemos em estudos de epilepsia de lobo temporal medial. Outras questões controversas, tais como dissociações simples e duplas, estudos de casos isolados versus estudos de grupos, bem como o método lesional são também examinados.

This article first reviews the cultural-historical and systemic approach to cognition, its mediated (semiotic) nature and its distributed network brain organization. Subsequently, methodological issues are examined including the use of this systemic and network approach in neuropsychological investigation, the concepts of single and double dissociation, single-case versus group studies, influential variables to be controlled in neuropsychological evaluations, and problems of brain-behavioral correlations using the lesion method.

The method for this review entailed a search of the PUBMED database for articles published up to 2010, which specifically addressed methodological issues and controversies in cognition disorders research. The following key terms were used, separately or combined: “neuropsychology methods”, “neuropsychological tests”, “cognitive tests”, “cognition research”, “double dissociation”, “lesion localization”. The main source for this article was the chapter “Research on cognition disorder: methodological issues” published by Damasceno BD in J. P. Tsai (ed.), Leading-edge cognitive disorders research, Nova Science Publishers, 2008.3

## Cognition: cultural-historical and systemic approach

The human mind comprises a complex activity involving interconnected mental and cerebral processes (its systemic structure), which represent the physical and social world by means of signs (its mediated, semiotic nature) and result from the internalization (or appropriation by the individual) of external actions and relations with things and persons (its cultural-historical origin).^1,2^ This new concept of mental “function” represents a considerable theoretical-methodological contribution to basic and clinical scientific cognitive research, since it takes into account the interfunctional relationships between task-relevant psychological variables, whose unique (independent) contribution to the task outcome can be determined by multivariable statistical analyses.^3^

**Cognition as a cultural-historical and mediated activity** – The interaction of the individual with external things and persons is mediated by signs (particularly of a linguistic nature). Man’s actions upon things and persons are preceded by mental actions, symbolic representations, programs, and projects. According to the seminal ideas of Vygotsky (1935/1978)^4^ and Leontiev (1981),^5,6^ in terms of their origin, mental actions derive from the appropriation (internalization) by the individual of external, practical actions and relationships with people and objects of the natural and cultural world. These external practices, particularly those of productive labor activity, develop under conditions of cooperation and social interaction among individuals, and are mediated by two kinds of instrument - material (artifacts, tools) and psychological (signs), which are social and cultural products as concerns both their origin (created and improved by countless generations of human beings) and their use (learned in the joint activity with other individuals). In the appropriation of the culture by the child (cultural learning) a relevant role is played by imitation (reproduction of intentions, objectives, and cause-effect relationships perceived by the child), by children’s collaborative activity with each other, and by the adult’s intentional teaching.^7^ In this process of appropriation, the primary and decisive role is played by the child’s practical actions with people and objects, that is, by the child’s own material (object) actions, since it is a question of constructing, not the image of the action, but the ideal action, the mind as action.^8,9^ The acquisiton of higher psychological functions (the specific human forms of attention, memory, and intellectual reasoning) is mediated by language (first external, then internal), particularly by the dialogic, argumentative activity of the child with other people. In this activity, the child develops its social cognition, in two senses:

[1] on the one hand, the control of others by the child in so far as he/she acquires a “theory” about others’ minds (“theory of mind”),^10^ attributing mental states to others, making inferences about their intentions, desires and beliefs, predicting their actions, and acting upon them based on this knowledge;
[2] on the other hand, the control of the child’s actions, attitudes, points of view and behavior by others, by society’s dominant ideology.^11^

**Cognition as a functional system** – Every mental act (object perception, problem solving) is carried out by a “complex functional system”,^1,12^ which has also been conceived as a “neurofunctional network”,^13^ “distributed parallel processing”^14^ or “multiple drafts model”.^15^ This insight on mental functions was first proposed by Hughlings Jackson,^16^ who conceived psychological functions as organized into different levels of complexity and abstraction (the voluntary, conscious; and the involuntary, automatic, unconscious).

The functional system or network comprises various basic operations (processes) organized in an assembly of interconnected brain regions, each region making its specific contribution to the functioning of the system as a whole. As conceived by Gazzaniga et al.,^17^ mental operations involve taking a representation as an input, performing some sort of process on the input, and then producing a new representation, or output. Thus, mental operations are processes that generate, elaborate upon, or manipulate mental representations.

Every mental act has a dynamic psychological structure and cerebral organization which changes from moment to moment insofar as each task or operation continually switches from one to the other. Each activity requires a different ensemble of cognitive operations suitable to achieve its objective, besides motivational, volitional, and emotional components. Its objective remains constant, but the methods or basic operations can vary.^18^ This plasticity also characterizes the process of acquisition of any new ability (e.g., car driving). At first, the structure of activity is expanded and requires a number of essential aids for its performance, but afterwards it gradually becomes condensed and converted into an automatic (motor) skill. Thus, in the initial learning phase, the activity requires numerous mental components (conscious intention, attention, working memory, language, visual, auditory, tactile-proprioceptive, and spatial perception) and various brain regions (prefrontal, posterior parietal, premotor, basal ganglia, sensory-perceptive, and so on). Later on, when skillful mastery is attained, the execution of the task becomes automatic, partly unconscious, resembling a kind of implicit knowledge (procedural memory) which involves fewer brain regions (premotor, motor, parietal, basal ganglia circuit).

## Neuropsychological investigation: methodological issues

The neuropsychological investigation can have various objectives:

[1] to analyze the patient’s symptoms by means of appropriate tests in order to diagnose an organic brain dysfunction and, by disclosing double dissociations, to establish which basic mechanism is impaired, and thus to diagnose the side and locale of brain lesion;
[2] to yield a baseline and profile of cognitive deficits for future comparisons; and
[3] in forensic neuropsychology, to predict the functional relationship between the patient’s performance on neuropsychological tests and his behavior in real-world settings.

This article shall chiefly address the first objective. Nowadays, the lesion site can be quickly and accurately diagnosed with computerized tomography (CT) or magnetic resonance imaging (MRI). However, conventional MRI may not show signs of lesion in sites where the neuropsychological analysis indicates there is a dysfunction, as typically occurs in the early phase of Alzheimer’s disease, when a finding of impaired retrieval and recognition may suggest an entorhinal-hippocampal neuronal loss.

### Systemic or network approach

At least three basic assumptions orient analysis of cognitive syndromes in Neuropsychology and Behavioral Neurology.^2,13^ These assumptions are not applied to the analysis of sensory-motor symptoms such as the loss of general sensation or movement in one half of the body (hemi-hypesthesia, hemiparesis) or the loss of vision in one hemifield (hemianopsia), which indicate lesion in their respective contralateral primary cortical zones or projection pathways.

- Each brain region, particularly those in convergence zones, contains the neural substratum (basic operation) of different complex functions and can thus belong to various partly overlapping neuronal networks.

As an example, the left inferior-posterior parietal region (Brodmann’s area 39) plays relevant role in seemingly disparate tasks such as assembling the parts of a chair, reproducing a model by means of multicolored cubes, orienting to right or left in a street or in one’s own body, subtracting 6 from 11, and understanding logical-grammatical structures that express spatial relationships (e.g., “the father’s brother”, “above”, “below”, “between”, “before”) or sentences with embedded relative clauses (“The boy *who is embraced by the brother of his father* is going to travel now”). All these different tasks require a basic operation, namely spatial reasoning, albeit at the physical-concrete or the abstract-symbolic level. Therefore, the neuronal networks of these tasks partly overlapped in this brain region.

– A lesion limited to one region impairs different complex mental functions, thus resulting in multiple symptoms or neuropsychological syndromes.

This postulate is derived from the former. In a patient with a lesion in the left inferior-posterior parietal region we usually have, not an isolated symptom, but a syndrome comprising constructive apraxia, difficulties with mental rotation of figures, acalculia, left-right desorientation, and difficulties understanding logical-grammatical constructions (Luria’s semantic aphasia).

– Different components of the same complex function can be impaired by lesions in different regions or in their interconnecting pathways.

Each complex activity (problem solving, story retelling) requires a set of different basic mental operations, particularly those processed in multimodal convergence zones such as the prefrontal (for planning, monitoring and correcting), parietal-occipital (for spatial reasoning), and entorhinal-hippocampal (for long-term explicit memorization). In the case of lesions, it does not suffice for the evaluating clinician to state that a complex function is impaired. Instead they should explain in what way this function is impaired and establish the subtype or profile of the cognitive syndrome, which varies according to which basic component is impaired.

Syndrome analysis by means of a comprehensive neuropsychological test battery and appropriate control conditions can help to disclose which basic component is impaired and, by inference, which brain structure is damaged. The tests should be selected so as to evaluate or measure the relevant components hypothetically involved in the complex function studied. The most crucial form of control is to compare the patient’s test performance to that of healthy individuals. This is a condition for ascribing the patient’s poorer performance to their brain lesion.

Besides a control group, control tasks are needed to determine if the patient’s cognitive or behavioral problem results from dysfunction of a particular component (e.g., loss of spatial reasoning or long-term memorization) or whether it is a consequence of a more widespread disturbance (attentional deficit, depression). Consider for example, the evaluation of memory problems of a patient with temporal lobe epilepsy. The patient complains of forgetting names of things, messages to be delivered to another person, and where they put their belongings (keys, eye-glasses). In this case, you suspect an impairment of declarative, episodic memory, and decide to evaluate it with appropriate tests for verbal and visual learning (word list, series of figures). The patient’s low scores on these tests can result either from a primary disturbance of episodic memory or from disorder of another component relevant to learning tasks, such as verbal fluency, visual perception, attention, and mood state. Therefore, these components (interfunctional relationships) need to be assessed by means of appropriate control tests, such as the WAIS-R digit span for attention,^19^ FAS or category (animals) for verbal fluency,^20^ Poppelreuter’s overlapping figures,^2^ Hooper visual organization,^21^ or form discrimination test^22^ for visual perception; and Beck’s scale for mood.^23^

### Single and double dissociations

Focal lesion of the associative cortex often affects several complex functions (e.g., constructional praxis, calculation, understanding of sentences with logical-grammatical structure), since each of these functions depends on the same basic operation (spatial reasoning) that is impaired. Nevertheless, the lesion leaves intact other functions that do not depend on this operation (e.g., speech fluency, understanding of melodies and simpler sentences, and learning of word list).

In this case, when a lesion to brain region A disrupts function X but not function Y, we have a single dissociation between the functions. The term “dissociation” refers not to the loss of association between two brain regions, but to the dissociated performance of a subject who performs extremely poorly on one task and at a normal or very much better level on another task. In a single dissociation, the difference in performance level on tasks X and Y does not mean that the two tasks require a different set of basic operations, since task X may simply be easier than task Y, which requires a greater amount of cognitive resources.^24^

More robust conclusions about brain function can be drawn by finding another patient with the reverse condition, that is, a lesion to region B, which disrupts function Y while leaving function X intact. A “double dissociation” can then be established, a concept introduced by Teuber,^25^ and further developed by Weiskrantz,^26^ Kinsbourne,^27^ and Shallice.^24^ With this concept, more specific inferences can be made about brain function even in functional neuroimaging studies of normal subjects. For example, consider two brain regions, A and B, and two experimental tasks, X and Y: during execution of task X, region A is active, but B is not, the opposite occurring during performance of task Y. Since one experimental manipulation has an effect on A but not on B and another on B but not on A, the conclusion can be drawn that these regions have different functional properties.^28^

Illustration: seeking double dissociations in the neuropsychological evaluation of medial temporal lobe epilepsy (MTLE) – Temporal lobe epilepsy is the most common partial epilepsy in adults, often associated with mesial temporal sclerosis (MTS), memory decline, and drug resistance, but presenting good postoperative outcome. The seizures usually arise from the medial temporal region (amygdala, hippocampus, parahippocampal gyrus). In the interictal period, these patients may present difficulties in memory, concentration, and reasoning, and may also manifest astheno-emotional, depressive and psychotic (rarely) disturbances.

The main challenge in neuropsychological investigations is establishing the side and site of temporal lobe dysfunction based on results of the testing alone, in the absence of clinical-electroencephalographic, MRI and/or single-photon emission computed tomography (SPECT) data. When the data from these three sources point to the same side and locale of brain dysfunction (epileptic focus and/or MTS), then we can make a safer surgical decision and predict good postoperative outcomes.^29,30^

Memory assessment is the most important component of the whole cognitive testing process in patients with MTLE, due to the relevant role of medial temporal lobe (MTL) structures in memory functioning and to the high prevalence of memory impairment in these patients. As emphasized by Milner^31^ and Jones-Gotman,^32^ memory testing should take into account the functional difference between the two temporal lobes: the left (dominant) temporal lobe mediates memory for verbal material, such as names, word lists or number sequences, while the right temporal lobe mediates memory for material that cannot be readily verbalized, such as faces, places, or abstract designs. Thus, in order to detect a double dissociation in the functions of both temporal lobes, Jones-Gotman^32^ proposed that:

[1] the test material should be highly polarized regarding its verbal versus non-verbal character (for example, list of abstract words versus abstract designs);
[2] the tests ought to be learning tasks, with repeated presentation of the material, so unimpaired individuals can improve with additional exposure to the material, increasing the difference between individuals with a true learning deficit and those who perform poorly on a first trial for other reasons; and
[3] the tests should be matched as regards the kind of memory activity they require (recall or recognition), varying only in terms of the verbal versus non-verbal nature of the material.

According to this author^32^, two of the most used tests (Wechsler Memory Scale and Rey Complex Figure) are not well polarized into their verbal and non-verbal material nor are they matched for the kind of memory activity involved, and therefore are unsuitable for comparisons between the hemispheres.

In summary, memory disturbance in MTLE is the result of interplay of the epileptic focus, the underlying brain lesion (MTS), and side effect of AEDs. Other influential variables are an earlier age at seizure onset, longer duration of epilepsy, and higher seizure frequency. MTS however, is associated with a family history of epilepsy, febrile seizures in infancy, and previous status epilepticus. The interplay is even more complex when considering memory as part of a network where it has interfunctional relationships with other psychological variables, such as mood state, attention, perception, executive function, and IQ. These variables can affect performance on memory tasks and need to be controlled and considered in a multiple regression (such as backward stepwise regression) or multivariable statistical analysis (such as ANCOVA), otherwise the finding of low memory scores cannot be explained by a memory specific impairment.

### Single-case versus group studies

The experimental method entails isolating and testing individual variables in order to verify possible cause-effect relationships or, at least, associations between events. In this regard, a case-control design is commonly adopted. To satisfy the homogeneity principle, all cases should have the same kind of lesion (e.g., infarction), and all cases and controls should be identical for all influential variables except that which is being studied. The problem is that brain lesions produced by natural diseases (stroke, multiple sclerosis, Alzheimer’s disease) are never exactly alike, varying from patient to patient in size, distribution, and mental-behavioral manifestations. This anatomical and behavioral variability among patients indicates that different functional subsystems are damaged, and creates difficulties when including these patients into a single lesion group (e.g., parietal or frontal). Furthermore, group studies may present practical difficulties. Due to the strict selection criteria, it may take a long time to collect enough patients, and even when the patients fulfill these criteria, they may not be in a state to be tested or may be unwilling to do so at the appropriate time.^24^

In view of these limitations to group studies, some authors^33^ have argued that single-case studies can contribute to better understanding of cognitive processes, by comprehensively analyzing the performance of individual patients and making comparisons across multiple cases. Damasio & Damasio^34^ also consider the ideal approach to be the collection and analysis of multiple single-case studies, particularly those based on high-level of sophistication of cognitive constructs and anatomical-neuroimaging resolution. In fact, the detailed study of individual cases can yield more information than large series (groups) in which many potentially interesting findings would not be detected in the statistical handling of the data. According to Gazzaniga et al.,^35^ the single-case study method affords powerful insights into the functional components of cognition, as exemplified by Caramazza’s study of patients with category-specific anomia. One of these patients showed a consistent and striking disability in naming fruits and vegetables, but no impairment in naming objects such as tools or furniture,^36^ thus supporting a category-specific organization of the mental lexicon.

The problem with single-case studies is their limitation for linking cognitive operations to neural structures, even in patients with unique cognitive deficits associated to a focal cortical lesion.^35^ It is difficult to know which affected region is correlated to the deficit, since the lesion usually involves several disparate cortical structures plus the underlying white matter, besides every patient has idiosyncrasies in cognitive-cerebral organization such that a lesion of the same locale, size and nature can produce different syndromes in different subjects. Even more controversial is the viewpoint of some cognitive psychologists who take into account exclusively occurrences in the cognitive system, without taking underlying cerebral processes into account. Their inferences are based solely on data collected from isolated single cases, without comparison to data collected from suitable control subjects, while their theoretical constructs may bear no relation to true psychological functions or less still to physiological cerebral functions.^37^ Comparison of patient performance to that of controls or a normative group is needed to avoid Type I errors. In many single-case studies, the sample size of the control or normative group is typically small (5 to 10 subjects), and Crawford & Garthwaite^38^ suggest an increase in control sample size to 20-30 in future single-case studies to achieve a marked effect on power and to reduce Type I errors.

Group studies however, can yield better insights into brain-behavior correlations. A method frequently used to establish the effective lesion site is pooling of a group of patients with a particular deficit, superimposing the positions of their CT or MRI lesions, and looking for a “hot spot”, that is for a site where all lesions overlap^24^ (but see limitations of this method on section on “brain-behavioral correlations: the lesion method”).

### Influential variables to be controlled

Neuropsychological assessment and particularly the interpretation of its findings is a challenging task, due to the complexity of functions evaluated and to the great number of variables involved. These variables concern the patient, their brain lesion, the tests employed, and testing conditions.

***Patient variables*** – Every patient has peculiar characteristics which influence test performance. Particularly age and education should always be considered when matching control subjects for cognitive studies. Cognitive abilities depend on age and they may not be fully developed in children and adolescents, or in those who have suffered deterioration in normal aging. Part of the age effect is due to cultural differences. Our cognitive-cerebral organization also depends on *educational and cultural experience*. Individuals who are illiterate or have a low educational level invariably experience difficulties on metalinguistic tests, especially those requiring pen and paper. This group has difficulties understanding test instructions, and show an attitude of suspicion or aversion towards the tests. *Handedness* should also be taken into account when comparing lesion groups. There can be left hemisphere dominance for language in right-handed and variable linguistic-cognitive hemisphere dominance in ambidextrous and left-handed subjects. As regards *gender*, the most consistent finding has been better performance by women in verbal fluency and by men on visual-spatial reasoning tasks. *Polyglotism* may also influence cognition, since certain peculiarities of each language (e.g., right-left versus left-right or up-down handwriting, phonologic versus ideographic type) engage specific cognitive-cerebral processing mechanisms. These differences lead to variations of syndromes in each language mastered by the same subject. *Personality, autobiography, affective and behavioral patterns, previous experience with the proposed test* or similar cognitive tasks such as checkers or chess playing, puzzle solving and word search can influence test performance and reaction to brain lesion. Personality changes may constitute the fundamental core of the resultant neuropsychological syndrome. Other influential variables are *motivation, interest, anxiety, restlessness, depression*, and *side effect of drugs*. An often neglected variable is the *circadian variation of cognitive functions*, performance on which is worse between 12 a.m. and 2 p.m. and after 23 hours.

***Lesion characteristics*** – Neuropsychological syndromes vary depending on site (brain region, focal versus multifocal or diffuse), size, nature (etiology), speed of installation, and age of lesion. With reference to speed of onset, for instance, multifocal and diffuse brain lesions can produce either a confusional state (delirium) or dementia, depending on their rapid (acute) or slow (chronic) course, respectively. Comparative studies should include patient groups of the same lesion age, since the clinical syndrome changes as the time passes (“syndromenwandel”),^39^ as typically happens with global aphasia evolving to Broca’s aphasia.

***Tests*** – Task performance is influenced by the kind of stimulus employed (visual, tactile, or acoustic-verbal) as well as their intensity and complexity. Also relevant are the presence (or otherwise) of previous instructions, the ecological validity of the test, and its metacognitive or artificial character. Ecologically-adapted tests, with tasks similar to those of everyday life, are easier to perform.

***Testing conditions*** – Other equally important variables to be controlled for are the examiner’s experience with the test, the patient’s acquaintance with the examiner, and the presence of incommodious light, temperature, or noise in the testing setting. Other “noises” to be avoided are anything that distracts the attention of the patient, a framed picture of fighting pirates on the wall for instance, or the examiner’s improper dress or moody look, and the examination table with too many things in disarray. This quiet testing environment as well as the control of most influential variables are crucial for establishing brain-behavior relationships, as recommended by Lezak.^20^ However, this “sterile environment” and the tests employed may be inadequate when the objective of neuropsychological evaluation is to predict (generalize) the patient’s behavior in noisy real-world settings within the home, workplace or community, hence the need to develop ecologically valid tests and testing situations.^40^

### Brain-behavioral correlations: the lesion method

Brain lesion secondary to natural diseases, surgical resection or experimental ablation in animals, is usually accompanied by functional disturbances in other interconnected and seemingly intact regions due to immediate changes in neurotransmission, excitation-inhibition balance, and blood flow (dyaschisis phenomenon of von Monakow),^41^ besides mass effect, changes in cerebrospinal fluid dynamics, secondary axonal degeneration, and sometimes septic complications and non-intentional damage to neighbouring structures. Other brain regions normally activated or inhibited by the injured region are denervated and may become hypo- or hyper-active. Disturbance of excitation-inhibition balance plus functional reorganization in other brain regions play a role in the resultant syndrome.^42^

Thus, to localize a lesion by neuroimaging does not imply localizing the same region as the symptoms (syndrome) or the impaired or lost function. Furthermore, natural lesions are often not well demarcated or limited to a precise neuroanatomical structure whose specific functional role is the target of investigation. In spite of these limitations, the lesion method based on clinical-anatomical correlations has been one of the greatest contributors to our knowledge of the cerebral organization of psychological processes, particularly before the imaging era.

Damasio & Damasio^34,43^ defined the following prerequisites for valid lesion-behavioral correlation:

[1] choice of appropriate neuropathological specimens and adequate timing for neuroimaging and concomitant neuropsychological testing;
[2] gathering of sufficient images for anatomical diagnosis; and
[3] interpretation of the group data, taking into account individual variations in neural-cognitive organization, age, sex, education, as well as psychological and social characteristics of the subjects.

According to these authors, the lesions most suited for behavioral and anatomical studies are:

[a] strokes, particularly non-hemorrhagic infarction at a chronic stage (more than three months old); followed by
[b] resolved herpes simplex virus encephalitis, and
[c] neurosurgical ablation of tumor or of epileptic focus.

In cerebral infarctions, particularly in the chronic phase, the abnormal signal area seen on CT or MRI corresponds to actual destruction of brain parenchyma, while in space-occupying lesions such as hemorrhage and infiltrating tumors, much of the abnormal area does contain functionally competent neurons. The problem with surgical removal of epileptic focus is that some patients may have widespread brain abnormalities due to long-standing seizure discharges, particularly in individuals with multifocal or bilateral epileptogenic activity. Other epileptic patients may have disorders of neuronal migration (e.g., micropolygyria), which sometimes go unseen on conventional MRI, requiring curvilinear reconstruction technique to be detected. The inclusion of such patients in lesion studies must be evaluated on an individual basis, giving preference to those which had a consistently unilateral epileptogenic focus prior to the surgery, and are in seizure-free state after a well delimited surgical resection (e.g., medial temporal lobe removal or amygdalohippocampectomy for treatment of medial temporal lobe epilepsy).

MRI lesion localization by looking for a “hot spot” in a group of patients with a particular deficit can be misleading if control patients are not used. A control group of patients without the deficit of interest is needed for safe conclusions, since the “hot spot” might reflect increased vulnerability of certain regions to injury (for example, because of their vasculature), rather than having any direct involvement with the disorder of interest.^44^ Lesion-behavior mapping (LBM) may focus on regions of interest – the ROI method, which can only identify patterns within predefined brain regions. Alternatively this can be performed by voxel mapping of the entire brain, with an independent statistical test conducted for each voxel, thus revealing critical brain regions associated with a given deficit without a priori assumptions. Various methods for voxelwise LBM have been developed including BrainVox,^45^ MRIcro,^46^ NPM,^47^ and Anatomo-Clinical Overlapping Maps (AnaCOM).^48^ In our Neuropsychology and Neuroimaging laboratories, we have used voxel-based morphometry (VBM) to correlate areas of brain atrophy to episodic and semantic memory performance in patients with amnestic mild cognitive impairment (aMCI), mild Alzheimer’s disease, and normal controls.^49-51^

In spite of many improvements in LBM methods, at least two main limitations remain:

[1] their “modularity” or “localization” assumption, considering the functional modules as having the same locations in different individuals, and neglecting that they change their function in response to damage to one area, while most brain damage is not limited by the boundaries of the underlying functional modules;^52^
[2] their low temporal resolution, which prevents the detection of functional (plastic) changes in other intact brain regions associated to the “syndromenwandel”, which follows the lesion. Many of these limitations of the lesion method have been partly overcome with functional neuroimaging, particularly with functional MRI (fMRI).

In fMRI studies, cerebral regions are activated by stimuli or tasks presented in blocked or event-related design, which result in vasodilation and increase in blood flow and in the ratio of oxygenated to deoxygenated hemoglobin, thus creating the BOLD (blood-oxygenation-level dependent) contrast, which is the basis for image construction. This image is a statistical map overlaid on a normalized anatomical MRI image, whose colors indicate the probability that the findings could occur under the null hypothesis. With fMRI we can look at the brain activity of healthy people and see every part of a neural network that is involved in a task or behavior, and thus can eliminate the problems of differential vulnerability, plasticity and disconnection associated with the lesion method.^44^ Notwithstanding these advantages, fMRI has limitations that should be taken into account when drawing conclusions about brain function on the basis of its results:^3,28,44,53,54^

[1] the fMRI “normalization” process can lead to errors, particularly in elderly stroke patients, since individual brains vary in their pattern of folds, size, overall shape and ventricle size, and all their images have to be matched to a common (normalized) template image;
[2] it has limited time resolution, since cortical neuronal responses occur within tens of milliseconds following a stimulus, while the first observable hemodynamic changes do not occur until 1 to 2 seconds later;
[3] it may present artifacts related mainly to magnetic susceptibility changes in the frontal and temporal lobes, which tend to be areas of interest in fMRI studies, particularly in patients with MTLE;
[4] it yields a great deal of activated areas thereby hampering interpretation, some of which may have no direct role in information processing; and
[5], as with other brain activation methods, fMRI determines the brain regions involved with a task, but does not reveal which areas are necessary or critical for performing the task, and therefore a balanced approach is to combine brain activation techniques (such as fMRI) with brain disruption technique such as LBM, as proposed by Rorden et al.^54^

## Conclusions

The human mind comprises a complex activity mediated by signs and involving various interconnected mental and cerebral processes. Research on cognition disorder must take into account this network structure of cognitive functions and their relationships with each other and with non-cognitive functions such as motivation, volition and emotion. Methodologically, the researcher has to consider the influence of task-relevant variables by means of suitable control conditions in order to disclose double dissociations, which may suggest the basic mental component impaired and by inference, the damaged brain region. In this kind of study, it is crucial to choose appropriate neuropathological specimens and adequate timing for neuroimaging and concomitant neuropsychological testing, and to interpret the group data taking into account individual variations in neural-cognitive organization such as age, sex, education, as well as psychological and social-cultural characteristics of the subjects. Both qualitative and quantitative methods in single-case and group studies are valid, depending on the objective of the research.
